# I don’t know-responses in the international trauma questionnaire caregiver-version: caregivers’ knowledge about children’s mental health

**DOI:** 10.1186/s40359-025-03229-3

**Published:** 2025-08-06

**Authors:** Brigitte Lueger-Schuster, Dina Weindl-Wagner, Andrea Zagaria, Moritz Russo, Irina Zrnić Novaković, Karin Zajec, Judith Noske, Alexander Haselgruber

**Affiliations:** 1 Unit of Psychotraumatology, Department of Clinical and Health Psychology, Faculty of Psychology, University of Vienna, Wächtergasse 1, Vienna, A-1010 Austria; 2 Department of Child and Adolescent Psychiatry and Psychotherapy Hinterbrühl, Federal Clinic Baden-Mödling, Hinterbrühl, Austria; 3 Department of Psychology and Cognitive Science, University of Trento, Trento, Italy; 4 Vienna Doctoral School in Cognition, Behaviour and Neuroscience, University of Vienna, Vienna, Austria

**Keywords:** Trauma, Children, Adolescents, PTSD, Assessment, Caregiver-assessment

## Abstract

**Background:**

To assess psychological problems in children having experienced traumatic events, multiple sources of information are gathered, including the caregiver’s report. Caregiver and child reports rarely accord. As there is no consensus why reports differ, this study investigated the caregivers’ knowledge on their child’s psychological symptoms, implementing an “I don’t know” option (IDK) in a measure on (complex) posttraumatic stress disorder– (C)PTSD.

**Methods:**

Data of 269 caregivers (including 70.19% biological parents and 11.15% other caregivers) and their children (*M*_*age*_ = 13.02, 55% female) were collected at entry assessments in a child psychiatry hospital. Standardized measures, including the International Trauma Questionnaire - Child and Adolescents Version (ITQ-CA), and a mirroring Caregivers’ Version (ITQ-CG) were used. Gender and age differences in IDK were investigated through Fisher’s Exact Tests and Mann-Whitney-U-Tests. Spearman correlations were computed between the total psychological outcomes as reported by children and caregivers. A Latent Class Analysis (LCA) was performed to identify different types of “Knowers” and “Don’t Knowers”.

**Results:**

43.49% of caregivers used at least one IDK response. IDK responses were unevenly distributed across (C)PTSD symptoms. There were no significant age or gender differences in children or caregivers regarding specific or total IDK. Social anxiety and suicidal tendencies were significantly associated with IDK. The LCA identified two caregiver-classes (“Knowers” vs. “Don’t Knowers”).

**Conclusions:**

The patterns of IDK underline the necessity of implementing this response option to provide clinicians with insights on important areas of psychoeducation for caregivers.

## Background

Exposure to at least one traumatic event (e.g. violence, sexual abuse) or the witnessing of a threatening event has been reported in two thirds of children and adolescents [1]. Trauma-exposed children and adolescents (henceforth referred to as “children”) have an increased risk of developing mental health problems, the most prominent being post-traumatic stress disorders (PTSD) and complex PTSD (CPTSD), disruptive behavior disorders, disorders due to substance use, anxiety disorders as well as depressive disorders [1–3]. Heterogenous symptoms and difficulties are associated with trauma sequalae in children [4] and may proceed into adulthood [5].

### Child- and caregiver reports in clinical practice

In clinical practice with children, clinicians use multiple sources in addition to the child’s self-report. One particularly important source of information is the child’s caregiver [6]. Caregivers can be natural, adoptive or foster parents, family members, or other legal guardians in charge of taking care of a child. Arguments for integrating external assessment of psychological symptomatology include gaining a broader picture of the symptomatology [7], the assumption that caregivers’ information may be more accurate [8], especially when considering factors of shame or suppression [9], and getting to know the caregiver’s perspective to better understand the caregiver– child relation [10].

Nevertheless, a low concordance between child- and caregiver reports has been found across various studies [11] and has even been deemed one of the most robust findings in clinical child psychology [12]. Multiple studies referred to the complexity of diverging results of caregivers’ and children reports in a broad range of areas and symptoms [13, 14] including trauma related symptomatology [15, 16]. A meta-analysis by Achenbach et al. [11] found higher levels of agreement between child and caregiver reports of externalizing compared to internalizing symptoms. The level of agreement has also been shown to vary, depending on the type of traumatic event a child has been exposed to [17]. In a study on complex trauma and sexual abuse in treatment seeking children, the concordance between caregivers’ and children’s report of symptoms was low, with caregivers reporting higher levels than children [18].

Skar et al. [17] showed that children reported higher rates of traumatic exposure across different age groups compared to their caregivers. A recent meta-analysis [19] further stated that age-appropriate criteria for PTSD were essential for identifying children who were in need of expert support. For caregivers it may be specifically complex to recognize the age-bounded expressions of trauma related symptoms [15, 17]. Differences in self- and caregiver reports can also indicate a caregiver’s missing health literacy. The concept of *mental health literacy* can be defined as “knowledge and beliefs about mental disorders which aid their recognition, management and prevention” [20] and has been found to generally be restricted in caregivers [21]. Moreover, the experienced trauma might affect differences in self- and caregiver reports [9].

Low concordance between child- and caregiver reports may also reflect issues in the child-caregiver relation. Child disclosure has been associated with a positive parent-child relationship as well as with increased parental knowledge both as perceived by the child and by the caregiver [22, 23]. Previous research has yielded inconsistent findings on the impact of informant/child gender on informant discrepancies. An early meta-analysis [11] found no significant differences between mothers’ and fathers’ rating, as well as caregivers’ or teachers’ ratings of boys compared to girls. In a consequent meta-analysis, Duhig et al. [24] reported discrepancies between father/mother reports to be greater regarding internalizing compared to externalizing symptoms. The child’s gender, however, did not have an impact on the correspondence between father and mother reports on behavioral problems.

Taking together, discussed reasons for the low concordance are manifold, ranging from informant characteristics and observability of attributes to different perception of informants [12, 25, 26]. As pointed out by De Los Reyes and colleagues [13] these differences can carry meaningful information and should not be discarded as mere measurement error. However, there are no definite answers regarding *who should report* and *how should be reported* about a child’s mental health condition in addition to the child itself to gain a maximum of information.

### State-of-the-art assessment of trauma in children and caregivers

Currently, there is one measurement instrument to assess trauma exposure and PTSD in children and caregivers consistent with the algorithm of the Diagnostic and Statistical Manual of Mental Disorders, Fifth Edition (DSM-5), the Child and Adolescent Trauma Screen (CATS [27]). The CATS has been validated for the use in children from age seven and above, including 15 items for traumatic exposure, 20 items for psychological reactions, and three items for psychosocial functioning, with an update for International Classification of Diseases (ICD-11), the CATS-2 [28]. Notably, the items were not altered accordingly, merely the scoring algorithm was adapted. A mirroring version for caregivers of children three to six years, and for ages seven and above exists [27]. However, this instrument does not address symptoms explicitly according to the ICD-11 conceptualization of disorders specifically associated with stress [29].

A reliable self-assessment tool for trauma specific symptomatology in children according to the ICD-11 is the International Trauma Questionnaire– Child and Adolescent Version (ITQ-CA; [14]). The ITQ-CA has been successfully implemented in studies, e.g. comparing DSM-5 and ICD-11 PTSD rates [30, 31] and has been validated in several languages [32, 33]. As stated earlier, for clinical practice and research in child clinical psychology and child psychiatry, caregiver reports are crucial, although valid measures of caregiver reports are rare. To fill this gap, the International Trauma Questionnaire– Caregiver Version (ITQ-CG; [34]) was developed, with an initial validation study recently published [35].

### The need for an “I don’t know” option

As discussed earlier, discrepancies between child and caregiver reports are the norm rather than the exception in child clinical psychology [12]. To better understand the cause of these discrepancies, further information should be collected when assessing children and their caregivers. One helpful source of information might be the addition of an „I don‘t know“ (IDK) option in the caregiver reports. This option could be clinically meaningful, since it indicates in which issues and with which frequency caregivers are unaware of the concrete psychological problems of their child. Providing an IDK option is, to this date, rare in psychological assessment. The existing literature examining IDK responses is mainly rooted in medical research (e.g. knowledge of risk of cancer; [36, 37]). The integration of an IDK option into a questionnaire needs to overcome several obstacles. For instance, its endorsement may be influenced by the relation between child and caregiver [38], gender perspectives [39], the parental response towards the child’s trauma [40], and the caregivers’ position in a child’s life [41], for example a parent or a caregiver from an institution [42].

The lack of an IDK option may minimize the caregivers’ ability to give a valid response and force them to choose a response that does not reflect the true observation of a child’s behavior [43]. On the other side, treating IDK responses as missings might undermine survey validity through the loss of information and a possible reduction of statistical power [36, 43]. Given that most of the caregivers do not have high mental health literacy, the inclusion of the IDK option represents a reasonable approach, since it does not force informants to respond to a question when not knowing the response. Moreover, possible associations between the parents’ IDK responses and the child’s mental health problems might be indicative for the child ‘s difficulties, as the absence of knowledge might contribute to the worsening of symptoms.

### Aims of the present study

The main objective of the present study was to gain new insights into caregivers’ (lack of) knowledge of their children’s mental health problems. In the newly developed trauma specific measure ITQ-CG, an IDK option was therefore implemented [35]. To the best of our knowledge, there is no study including an IDK option for caregivers in a trauma assessment. The present study was thus conceptualized as an exploratory study aiming to tackle challenges and opportunities related to the implementation of an IDK option in this specific setting.

We explored the frequency of IDK responses in each ITQ-CG item and as a function of the child’s gender and age. The study compared different types of caregivers (e.g., parents vs. other caregivers). Further, we explored the relevance of parents not knowing symptoms in children’s psychopathology, by correlating IDK responses with different psychological symptom outcomes, specifically for PTSD and CPTSD symptoms. Finally, the study aimed to identify distinct types of response tendencies among the caregivers.

## Methods

### Participants and procedure

Data utilized in this investigation was collected as a part of a clinical screening of every new patient treated at the Department for Child and Adolescent Psychiatry and Psychotherapy in Hinterbrühl, situated at the Federal Clinic Baden-Mödling in Austria between January 2023 and January 2024. The catchment area of the department includes about 773,264 inhabitants providing psychiatric treatment for acute crisis, inpatient and outpatient units [44]. Caregivers of 326 newly presented patients aged six to 17 completed a standardized set of clinical measures rating the symptom burden of the children. No specific information about the caregivers except their relation to the children was collected. Additionally, children aged seven to 17 years were asked to fill out self-report questionnaires. Inclusion criteria required the patients to be aged between six and 17 years for caregiver-reports and possess sufficient proficiency in the German language. All patients and their caregivers gave written informed consent beforehand.

Approval for the study was obtained from the ethical review board of the University of Vienna (#00919) and the Government of Lower Austria (GS4-EK-4/870–2023).

### Measures

The *International Trauma Questionnaire– Caregiver version* (*ITQ-CG*; [34, 45]), was adapted from the ITQ-CA [46], retaining the original formulations, number of items, and scoring scheme. The only modifications were addressing caregivers as informants by adding “my child” to all items, similar to adjustments made by researchers in other PTSD measures [27], and including an IDK response option for all PTSD and the CPTSD-specific disturbances in self-organization (DSO) symptom items, to acknowledge that caregivers might not know some of their children’s mental health-related problems. This resulted in a 22-item caregiver-report measure designed to assess ICD-11 PTSD and CPTSD symptoms in children aged 6–17 years. Six items assess the three clusters of PTSD (re-experiencing, avoidance, and threat), with two items capturing **re-experiencing** (RE 1, RE 2), **avoidance** (AV 1, AV 2), and **threat** (TH 1, TH 2), respectively. Similarly, six items assess the three clusters of DSO (affective dysregulation, negative self-concept, disturbances in relationships), with two items capturing **affective dysregulation** (AD 1, AD 2), **negative self-concept** (NSC 1, NSC 2), and **disturbances in relationships** (DR 1, DR 2), respectively. These 12 items are listed in Table 4. In addition, caregivers rate how much their children were bothered by each symptom in the past month on a 5-point Likert scale ranging from 0 (“never”) to 4 (“almost always”), plus the IDK options. Five items measure functional impairment due to PTSD and DSO on a binary scale. In the present study, internal consistency was good, with Cronbach’s alpha values of 0.82 for PTSD and 0.79 for DSO dimensions.

The *International Trauma Questionnaire– Child and Adolescent Version* (*ITQ-CA*; [46]) was used to assess ICD-11 PTSD and CPTSD in children through self-report. The items and scoring are the same as in the ITQ-CG, except for the absence of the IDK option and for the addressed informant. Internal consistency was very good, for both PTSD (α = 0.81) and DSO (α = 0.85). A previous study examining PTSD and CPTSD symptomatology in children exposed to potentially traumatic experiences [47] showed comparable internal consistency for PTSD (α = 0.79) and for DSO (α = 0.86) subscale scores.

The *Child and Adolescent Trauma Screen (CATS) checklist* [27] was utilized to evaluate lifetime trauma in children. Exposure to potentially traumatic events is measured using 15 dichotomous items. In our study, both the self-report and the caregiver-report version were administered.

The *Short Mood and Feelings Questionnaire* (*SMFQ*; [48, 49]) is a 13-item tool designed to assess symptoms of depression in children. Caregivers or children rate each statement if it is true for them on a 3-point Likert scale, ranging from 0 (“not true”) to 2 (“true”). In our study, the SMFQ was administered as self-report and caregiver-report version [48, 49]. The measure demonstrated very good internal consistency in the caregiver-report (α = 0.84) and in the self-report (α = 0.89).

The *Screen for Child Anxiety Related Disorders* [50] is a 41-item instrument used to assess symptoms of anxiety disorders in children, as reported by their caregivers. It consists of five subscales (reliabilities in the current sample are noted in round brackets): panic disorder/somatic symptoms (α = 0.87), generalized anxiety disorder (GAD; α = 0.85), separation anxiety (α = 0.78), social anxiety disorder (α = 0.84), and school avoidance (α = 0.78). Caregivers rate the child’s recent anxiety symptoms on a 3-point Likert scale, ranging from 0 (“not true or hardly ever true”) to 2 (“very true or often true”). A previous study on help seeking adolescents [51] yielded slightly higher results for internal consistency ranging from α = 0.88 to α = 0.89.

The *Pediatric Symptom Checklist* (*PSC-17*; [52]) is a 17-item measure to assess emotional and behavioural problems. It was administered as caregiver-report [52] and self-report [53]. It comprises three subscales to assess attention problems (α = 0.73 by caregivers; α = 0.76 by children in the current sample), internalizing (α = 0.81 by caregivers; α = 0.79 by children in the current sample) and externalizing problems (α = 0.76 in caregivers; α = 0.65 in children in the current sample). The internal consistencies were comparable to those of a previous study on a general paediatric population [54]. Caregivers or children indicate on a 3-point Likert scale ranging from 0 (“never”) to 2 (“often”) how often the described problems are present.

The *Adolescent Dissociative Experiences Scale*, short version (*ADES*-8; [55]) consists of eight items inspired by the “taxon” version of the adult DES [56] describing symptoms of derealization, depersonalization, and compartmentalization [57]. It was administered via self-report: children rated how much they agree on a 11-point scale, indicating the frequency with which they perceive dissociative experiences occurring, ranging from 0 to 100%. Internal consistency in the current sample was good (α = 0.86) and thus higher than the internal consistency of α = 0.77 reported in the validation study by Martínez-Taboas et al. [55].

The *Generalized Anxiety Disorder* scale, adolescent version (*GAD-7*; [58]) is a 7-item self-report partially based on the Diagnostic and Statistical Manual of Mental Disorders, Fourth Edition (DSM-IV; American Psychiatric Association [APA]; [59]) criteria for GAD. It assesses the frequency of symptoms over the past two weeks. For each symptom, the following response options are provided: “not at all”, “several days”, “over half the days”, and “nearly every day”, which are scored as 0, 1, 2, and 3, respectively. Internal consistency in the current sample was good (α = 0.84). A previous study on Finnish adolescents [60] found an internal consistency of α = 0.91.

Item Nr. 9 of the *Beck-Depression-Inventory II* [61] was used to assess whether suicidal thoughts, ideas or plans were present in the children, both through a self-report and a caregiver-reported formulation [62]. The predictive value of using this item was reported in a number of studies [63] and its validity for use in children is well established [64, 65]. The items range from 0 (“I do not have any thought of killing myself”/ “My child does not have any thoughts of killing themselves”) to 3 (“I would kill myself If I had the chance”/ “My child would kill themselves if they had the chance”).

### Data analysis

All analyses were performed with the software R (Version 4.3.2; [66]). First, descriptive statistics for all ITQ-CG items were calculated, including *M* (*SD*), and examining an overall proportion of IDK responses for each item. A summative variable of IDK response was also created, that is the amount of IDK responses selected by each caregiver (i.e. providing one IDK response in total would be equal to 1, providing two IDK responses in total would equal to 2, etc.). That way, the “IDK” may be seen as a count variable. The distribution of the summative IDK variable was also displayed graphically.

Second, specific gender differences, also considering IDK responses, were examined item-wise through Fisher’s exact tests. Analyses of the IDK summative score in general and depending on age, were calculated with Mann-Whitney-U-tests (because of missing normal distribution; [67], p. 157–164). Third, differences between different types of caregivers were calculated with Fisher’s exact tests. Fourth, the relation of IDK responses (summative scores) and different psychological symptom outcomes (PTSD, depression, anxiety, suicidal tendencies, internal and external symptomatology) were investigated with Spearman correlation, due to missing normal distribution of data. Age was also investigated to the “know” vs. “don’t know” status, both specifically for each item and overall, using Mann-Whitney-U-tests (due to missing normal distribution).

Finally, Latent Class Analysis (LCA) was performed to identify homogenous classes of multivariate categorical data, aiming to investigate if there were discernible “types” of caregivers‘ response tendencies. The ITQ-CG responses on all 12 items were dichotomized. The 0,1,2,3,4 options were grouped under the same value and 5 (indicating “I don’t know”) was coded as the other value. Subsequently, the 12 ITQ-CG items were used as binary indicators to determine the optimal number of classes based on caregiver responses (knowing vs. not knowing). Commonly used fit indices to choose the optimal number of classes are the Akaike Information Criterion (AIC) and Bayesian Information Criterion (BIC) whereas lower values indicate better fit. The LCA was conducted with the poLCA package [68]. Models with 2 to 6 classes were analyzed, and their fit indexes and statistics, including negative log likelihood, AIC, BIC, G²w, and χ², were examined. Additionally, the Lo-Mendell-Rubin adjusted likelihood ratio test (LMR test; [69]) was conducted to compare models with increasing numbers of classes. A non-significant result (*p* >.05) indicates that the model with one less class should be accepted. However, in selecting the model, we will prioritize two criteria: the first and most important is the model interpretability [70]. Among the fit indices, particular attention will be given to the BIC criterion, which has been demonstrated to be arguably the most reliable in mixture models [71], as its purpose is to identify the true model rather than to predict new data [72].

## Results

### Descriptives

From 326 data sets, 57 cases were excluded (45 patients had more or equal to 50% missings in the ITQ-CG; 11 had more or equal to 50% missings in the CATS questionnaire; one person did not provide sociodemographic characteristics). Fisher’s Exact Tests and t-Tests revealed no significant differences between excluded and included participants regarding gender, caregiver type, age and previous stay at a psychiatric care unit. The final caregiver-report sample encompassed assessments of *N* = 269 children.

The mean age of children in the final caregiver-report sample was 13.03 years (*SD* = 2.94), with 119 boys (44.24%), 146 girls (54.27%) and four missing values regarding gender (1.49%). Distributions of living situations as well as current schools are listed in Table 1. Sixty-eight patients (25.27%) had been patients in a psychiatric facility before, 106 (39.40%) took medication regularly, 214 (79.55%) were under psychotherapeutic treatment. Caregiver-reported symptom levels of children’s different mental health conditions are summarized in Tabel 2.

With regard to the composition of caregivers compiling the questionnaire, 139 (51.67%) were natural mothers, 38 (14.12%) were natural fathers, 12 (4.40%) were natural mother and father compiling the questionnaires together, 30 (11.15%) were other caregivers, specifically: assigned caregiver of foster care institution (*n* = 12), foster mother or father (*n* = 6), stepfather or stepmother (*n* = 6), adoptive father (*n* = 1), grandparents (*n* = 3) and other type of caregiver (*n* = 2). The remnant 50 (18.58%) types of caregivers were missing. For most of the analyses, the types of caregivers were recoded as natural mother, natural father, or other type of caregiver.

To investigate associations of caregivers’ IDK responses with children’s self-reported psychological outcomes, a subsample of children’s self-evaluation was matched with the respective caregiver. As not all children completed the self-report questionnaires, this *Self-report sample* consisted of *n* = 185 patients. Sociodemographic data of this subsample is displayed in Table 1, symptom burden as reported by the children of the subsample is displayed in Table 2.

**Table 1:** Descriptive statistics for children’s sociodemographic variables

	CG-report sample				Self-report sample	
Variable	*M* (*SD*)		Range		*M* (*SD*)	Range
Age	13.03 (2.94)		6–17		13.46 (2.59)	7–17
Siblings	1.48 (1.12)		0–6		1.48 (1.18)	0–6
Variable	*n*		% ^a^		*n*	% ^a^
Gender						
Female	146		54.27		107	57.80
Male	119		44.24		73	39.50
Other					1	0.50
Current school						
Elementary school	27		10.11		14	7.57
Variable	n		% a		n	% a
Middle school	65		24.34		38	20.54
Gymnasium & Vocational Highschool	81		30.34		80	43.24
Special needs school	14		5.24		7	3.78
Occupied or in search of work	18		6.74		12	6.49
Other ^b^	60		22.47		34	18.38
Living situation						
With both natural parents	110		42.20		92	49.73
With one natural parent	111		41.57		72	38.92
Other Caregiver	12		4.49		4	2.16
Institution	15		5.62		9	8.90

Note. CG = Caregivers

^a^ Percentages of missing responses are not listed to improve readability unless stated otherwise

^b^ Missings, alternative schools and unspecific responses

**Table 2:** Descriptive statistics of children’s caregiver -reported and self-reported symptom burden

	CG-report		Self-report	
	*M* (*SD*)	Cut-Off	*M* (*SD*)	Cut-Off
Depression	12.78 (6.35)	9	14.09 (7.27)	5
GAD	8.04 (4.63)	8	11.56 (5.59)	11
Dissociation	*Not assessed*		2.43 (2.23)	4
	% diagnostic requirements met		% diagnostic requirements met	
PTSD ^a^	8.80		8.57	
CPTSD ^b^	17.62		25.70	

Note. CG = Caregivers, GAD = Generalized Anxiety Disorder, (C)PTSD = (complex) Post Traumatic Stress Disorder. Cut-Offs are based on the validation study of the SMFQ [49] for depression, a study on the psychometric properties of the SCARED [50] for CG-reported GAD, a validation study of the GAD-7 [58] for self-reported moderate levels of GAD and a validation study on the ADES-8 [55] for clinically relevant dissociative symptoms. The percentages of diagnostic requirements met were calculated based on the diagnostic criteria for (C)PTSD according to the validation study of the ITQ-CA [45]

^a^ Includes all patients who met symptom criteria for PTSD but not DSO

^b^ Includes patients who met symptom criteria for PTSD and DSO

### Frequency of IDK responses

**Fig. 1:**
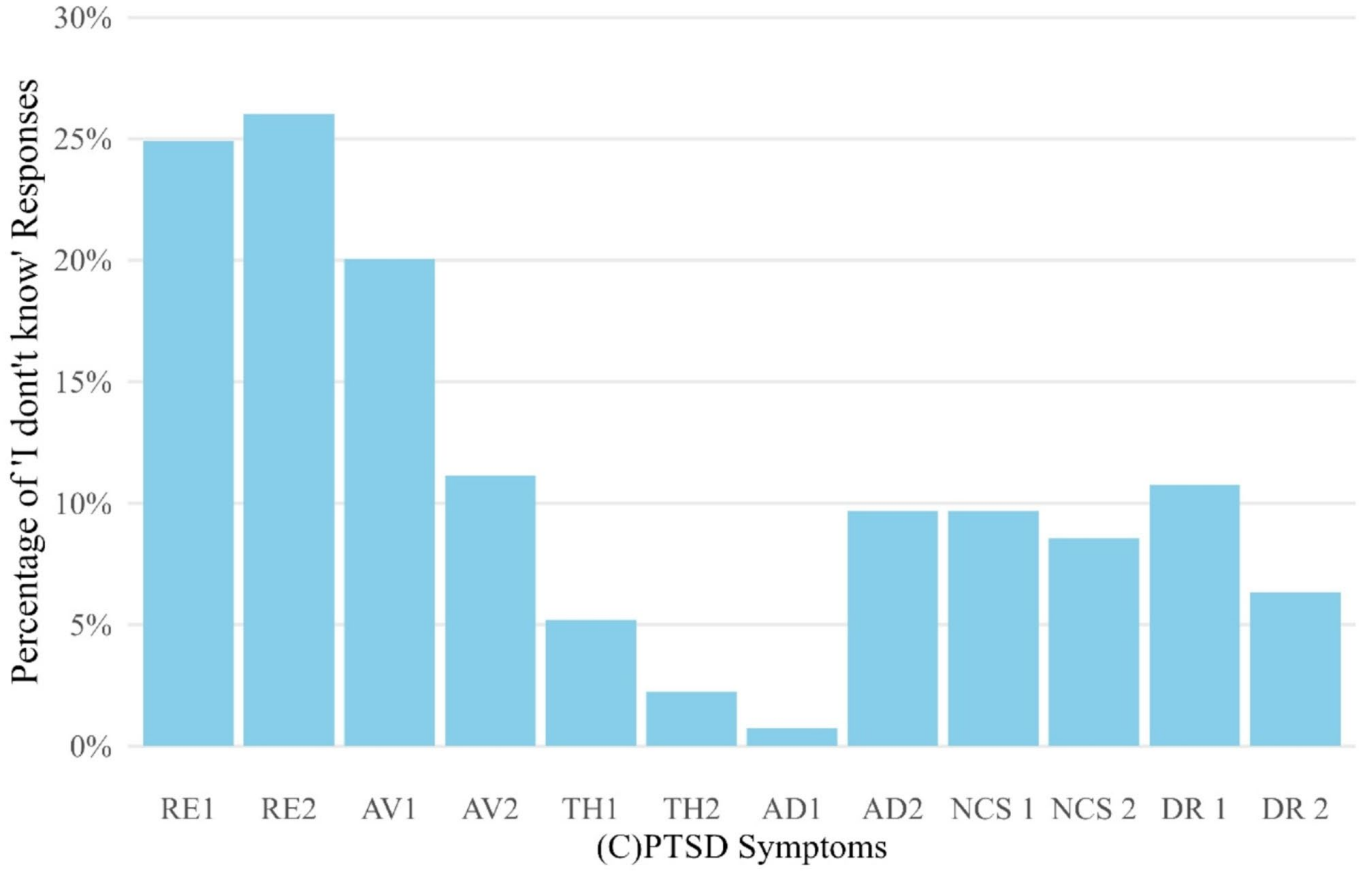
Percentages of IDK responses across the ITQ-CG items

The IDK percentages were unevenly distributed across the ITQ-CG items, with percentages being approximately ¼ of the total in each of the first three items (RE 1: 24.91%, RE2: 26.02%, AV 1: 20.07%), 11.15% for the fourth item (AV 2), almost null for items TH 1, TH 2, and AD 1, and about 10% in each of the remnant items (AD 2: 9.67%, NSC 1: 9.76%, NSC 2: 8.55%, DR 1: 10.78%, DR 2: 6.32%). A detailed numerical account of the IDK percentages across the sample is presented in Table 3. Figure 1 depicts distributions of IDK responses across items assessing (C)PTSD symptoms.

**Table 3:** Values (absolute and percentages) of IDK selected

Numbers of IDK selected	Absolute number of respondents	Percentage number of respondents
0	126	46.84
1	30	11.15
2	32	11.90
3	16	5.95
4	22	8.19
5	5	1.86
6	10	3.72
7	2	0.74
8	1	0.37
Missing	25	9.29

Note. Missing values refer to the participants who selected neither one of the options on the Likert scale (ranging from 0 = “never” to 4 = “almost always”) nor the IDK option in at least one of the questions

**Fig. 2:**
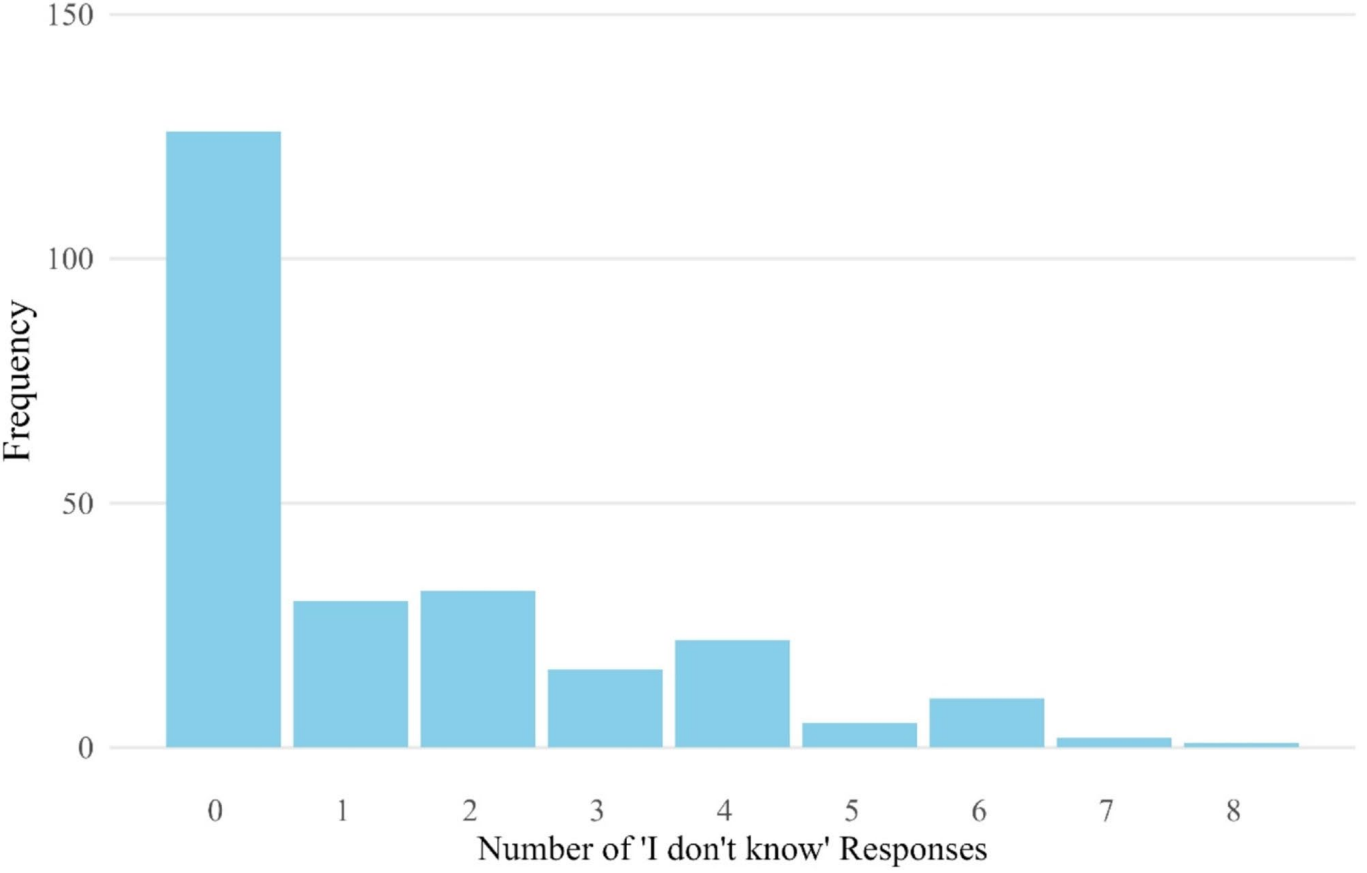
Histogram of raw frequencies of different IDK counts

The summative IDK variable presented a highly right-skewed distribution (Fig. 2). In our high-burden sample, a larger part of caregivers declared to know the children’s symptomatology for all items; specifically, about half of the caregivers (46.84%, *n* = 126) never used the IDK responses (Table 3). Some caregivers used it for one or two items (*n* = 30 and *n* = 32, respectively, that is 11.15% and 11.90%), with a declining proportion for using more IDK responses. However, a large part of the sample used at least one IDK response (43.49%, *n* = 118). Note also that a substantial percentage (9.29%) was also considered as “missing” in this regard because they had at least one missing value in the ITQ responses so the precise total count of IDK could not be computed.

### IDK responses based on child’s age and gender, and caregiver type

Fisher’s exact tests found no significant relationship between children’s gender and IDK in specific items (Table 4). No significant relationship was also found between the IDK summative score and child’s gender overall (W = 6666.5, *p* =.302). Even though not significantly, it may be worth noting that caregivers were more likely to report “IDK” for female children in comparison to male children for the PTSD items, while, on the contrary, caregivers were more likely to report “IDK” for male children in comparison to female children for the DSO items (Table 4).

Natural mother and fathers also did not differ significantly both in specific items (Table 1) and overall (W = 2168.5, *p* =.937). There was also no effect between natural parents vs. other types of caregivers both in specific items (Table 4) and overall (W = 2332, *p* =.8166). Eventually, we did not find any crosswise effect between parents and same-sex or other-sex offspring neither in specific items or overall, for mothers (W = 1628.5, *p* =.342) and fathers (W = 135.5, *p* =.567).

Mann-Whitney-U-tests showed that the only significant difference in age regarding specific items could be observed in item RE 1, with caregivers more frequently selecting the IDK option in older children (13.79 vs. 12.79, *U* = 7844, *p* =.02 see Table 1). All other age comparisons were not significant. A Spearman correlation between age (in years) and summative IDK score suggested a positive, but not statistically significant relationship, *r* (242) = 0.07, *p* =.233.

**Table 4:** IDK descriptive statistics and differences across children’s and caregivers’ gender and type

Items	M (SD)	Total % IDK	Age (mean IDK vs. mean IK)	Relative IDK % CA gender (male vs. female)	Relative IDK % CG gender (natural father vs. natural mother)	Relative IDK % CG type (natural parents vs. other)	Relative IDK % CA gender across mothers (male vs. female)	Relative IDK % CA gender across fathers (male vs. female)
Having upsetting dreams (RE 1)	0.95 (1.17)	24.91	13.79 vs. 12.79*	20.17 vs. 28.77	24.32 vs. 27.41	27.72 vs. 26.67	17.64 vs. 32.92	25 vs. 23.52
Having powerful images and memories (RE 2)	0.87 (1.20)	26.02	13.54 vs. 12.80	20.17 vs. 30.82	32.43 vs. 27.21	28.65 vs. 24.14	21.15 vs. 30.48	20 vs. 47.05
Avoiding internal reminders (AV 1)	1.43 (1.43)	20.07	13.31 vs. 12.80	16.81 vs. 23.29	19.44 vs. 21.32	22.28 vs. 17.24	15.38 vs. 25.60	15 vs. 23.52
Avoiding external reminders (AV2)	1.10 (1.42)	11.15	13.66 vs. 12.87	10.92 vs. 11.64	2.7 vs. 15.56	12.50 vs. 10.71	13.46 vs. 17.28	0 vs. 5.8
Being ‘super-alert’, watchful or on guard (TH 1)	1.03 (1.36)	5.20	12.85 vs. 13.03	3.36 vs. 6.85	2.7 vs. 7.41	6.52 vs. 0	3.84 vs. 9.87	0 vs. 5.8
Feeling jumpy or easily startled (TH2)	0.98 (1.22)	2.23	13.83 vs. 12.96	2.52 vs. 2.05	2.7 vs. 2.19	2.15 vs. 3.45	1.92 vs. 2.43	5 vs. 0
Long time to calm down when upset (AD 1)	2.51 (1.33)	0.74	13 vs. 13.03	0 vs. 1.37	2.7 vs. 6.52	1.09 vs. 0	0 vs. 1.2	0 vs. 6.25
Feeling numb or emotionally shut down (AD 2)	1.80 (1.41)	9.67	12.57 vs. 13.07	10.92 vs. 8.9	10.53 vs. 7.91	8.99 vs. 13.23	5.6 vs. 9.6	9.52 vs. 11.76
Feeling like a failure (NSC 1)	1.68 (1.30)	9.76	12.88 vs. 13.04	11.76 vs. 7.53	10.53 vs. 6.52	7.98 vs. 13.33	3.8 vs. 7.2	19.04 vs. 0
Feeling worthless (NSC 2)	1.27 (1.33)	8.55	13.43 vs. 12.98	10.08 vs. 7.53	10.53 vs. 5.04	6.35 vs. 16.67	5.6 vs. 4.8	9.52 vs. 11.76
Feeling distant or cut-off from people (DR 1)	1.56 (1.33)	10.78	13.06 vs. 12.97	13.45 vs. 8.9	13.16 vs. 11.76	1.83 vs. 3.33	12.23 vs. 7.4	14.28 vs. 11.76
Finding it hard to stay emotionally close to people (DR 2)	1.55 (1.38)	6.32	13.58 vs. 12.97	8.40 vs. 4.11	7.89 vs. 8.09	8.06 vs. 6.67	11.53 vs. 4.93	14.28 vs. 0

Note. CA = Children; CG = Caregivers; IDK = “I don’t know” responses, IK = “I know” responses (i.e. 0,1,2,3,4). The first six items were used to assess three clusters of PTSD, namely: re-experiencing (RE 1 and RE 2), avoidance (AV 1 and AV 2), and threat (TH 1 and TH 2). The next six items were used to assess three clusters of DSO, namely: affective dysregulation (AD 1, AD 2), negative self-concept (NSC 1, NSC 2) and disturbances in relationships (DR 1, DR 2). As the sample is biased in terms of absolute numbers regarding CA and GC gender, relative percentages are reported instead of absolute ones, in order to have an intuitive grasp at the proportions tested by Fisher’s Exact tests. None of Fisher’s exact tests yielded significant results (*p* <.05)

*p <.05

### IDK differences in psychological outcomes

To better understand the relationship between children’s symptom burden and caregivers’ knowledge, the summative IDK score was correlated with all psychological outcomes assessed in the study. None of the psychological outcomes showed a statistically significant relationship with the summative score of the IDK in the caregiver-reported questionnaires - except for social anxiety and suicidal tendencies, exhibiting a small (≈ 0.15) significant positive correlation. The specific Spearman rho values are listed in Table 5.

**Table 5:** Correlations of psychological outcomes reported by the caregiver with the summative IDK score

Variable	Summative IDK score
PSC externalizing subscale	− 0.01
PSC internalizing subscale	0.11
PSC attention subscale	0.01
GAD	− 0.01
SCARED panic/somatic	− 0.04
SCARED separation	− 0.06
SCARED social anxiety	0.17**
SCARED school avoidance	0.08
SMFQ depression	0.06
BDI suicidal ideation	0.15*
ITQ CG PTSD	− 0.03
ITQ CG DSO	0.09
CATS number of traumatic events	0.01

* *p* <.05. ** *p* <.01

Regarding the self-reports, none of the psychological outcomes showed a statistically significant relationship with the summative score of the IDK. The specific Spearman rho values are listed in Table 6.

**Table 6:** Correlations of psychological outcomes reported by the child with the summative IDK score

Variable	Summative IDK score
PSC externalizing subscale	0.06
PSC internalizing subscale	0.03
PSC attention subscale	0.07
ADES dissociation	0.06
GAD	0.05
SMFQ depression	0
BDI suicidal ideation	0.02
ITQ CA PTSD	0.08
ITQ CA DSO	0.06
CATS number of traumatic events	0.07

* *p* <.05. ** *p* <.01

### “Knowers” versus “don’t knowers”

Table 7 depicts the fit indexes for the LCA model.

**Table 7:** Fit indexes LCA

Model	LogLikelihood	AIC	BIC	G Square	Chi Square	Entropy	LMR test *p* value
**2 Classes**	-877.83	1,805.66	1,895.52	377.94	6,440.30	0.82	.
3 Classes	-845.49	1,766.99	1,903.59	328.32	3,556.54	0.83	0
4 Classes	-826.21	1,754.41	1,937.74	287.46	3,554.03	0.89	0.00004
5 Classes	-811.35	1,750.70	1,980.76	259.83	1,360.40	0.82	0.0088
6 Classes	-798.19	1,750.37	2,027.17	231.30	1,222.56	0.85	0.0239

**Fig. 3:**
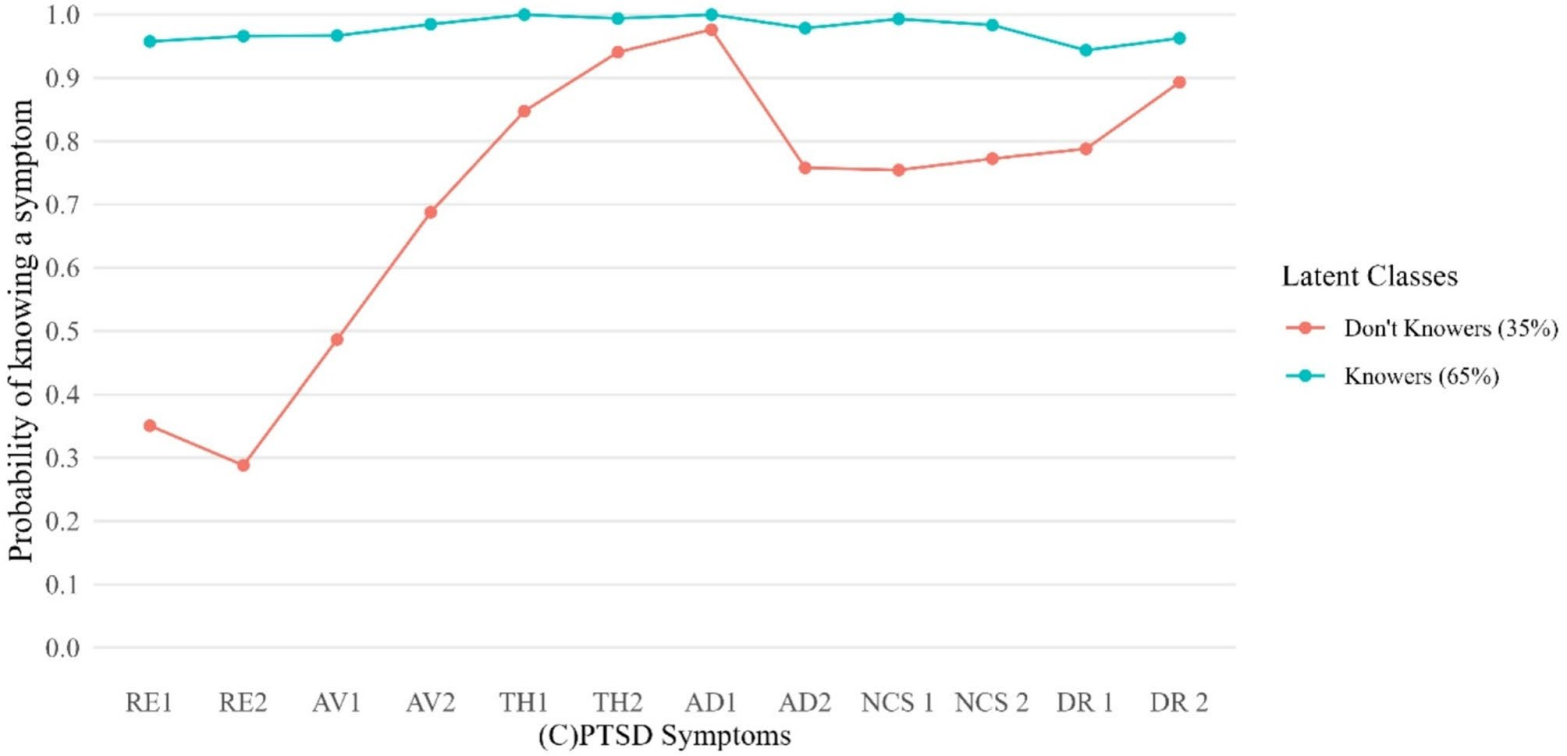
Profile plot of the 2 classes LCA model

The BIC indicates the best fit for the 2-class model, while the AIC index suggests that the 5- or 6-class models might be good candidates as well. The LogLikelihood is never significant, although it is the lowest among those indicated from the 5-class to the 6-class model. Consistent with what was previously stated in the methods, we prioritized the 2-class model, which had the lowest BIC and was clearly interpretable. The model can be viewed in Fig. 3. The y axis represents the probability of knowing the symptoms.

As illustrated, the figure closely reflects the simple percentage graph of the IDK responses (Fig. 1); high on the first three items and relatively compromised on certain DSO items. The LCA model, in this case, does not suggest any particular taxonic pattern among caregivers. Regarding the composition of the class, most caregivers (65%) are predicted to know very much about their children, while 35% are predicted to not know the answers to the first PTSD items. The LCA model thus seems to estimate a larger percentage of “Don’t Knowers” in respect to the simple raw frequencies (Fig. 1; Table 3).

## Discussion

The present study explored the caregivers’ (lack of) knowledge regarding their children’s mental health problems, using the recently developed caregiver-report measure for PTSD and CPTSD symptoms in childhood and adolescence (ITQ-CG). We examined the frequency of IDK responses and compared these responses based on child’s age and gender, and the type of caregiver (e.g. parents vs. other caregivers). Furthermore, we found significant associations between IDK responses and some psychological outcomes and identified distinct groups of “Knowers” and “Don’t Knowers”.

### Frequency of IDK responses

In our study we showed that roughly half of the caregivers (46.84%) never endorsed the IDK response, about 11% used the option for one or two items while less caregivers used it for three or more items. However, a large part of the sample (43.49%) used *at least* one IDK response. This points to the fact that a considerable number of caregivers may not have accurate and thorough knowledge of their children’s overall mental health state, especially with respect to certain symptoms.

### IDK responses based on child’s age and gender, and caregiver type

When looking into specific items, caregivers of older children reported *less* knowledge about children’s upsetting dreams than caregivers of younger children. This finding goes in line with past research indicating that children communicate less with their caregivers about their mental health issues, when they grow up [11]. Adolescence typically includes a greater autonomy from family, more personal independence, and a greater importance of peers [73].

Gender aspects for both children and caregivers did not show any statistically significant relation, though a tendency for using slightly more IDK responses for girls was observed for PTSD items. On the contrary, for the DSO items, IDK responses were endorsed slightly more for boys, which is in contrast to a study showing that earlier puberty mediates the link between trauma and externalizing problems in males [74]. Notably, our observations should be interpreted with caution, since the tested gender differences were not statistically significant. Moreover, no statistically significant differences in the use of IDK responses were shown between mothers and fathers, nor between natural parents and other types of caregivers.

### IDK differences in psychological outcomes

The only significant but small associations regarding psychological outcomes were found between the total IDK count and social anxiety, and between the total IDK count and suicidal tendencies as reported by the caregiver. No other psychological outcome exhibited a significant correlation with IDK responses. These initial findings suggest that caregivers who estimate their child’s symptoms of social anxiety and suicidal tendency to be high have, on average, less knowledge about their children’s mental health problems. In a study with help seeking adolescents it was reported that social anxiety and suicidal ideation were mediated via loneliness [75]. Loneliness might suggest a poor child-parent relationship and/or a weaker family connectedness, which might partly explain the lack of knowledge in parents, who consider fearfully that their children suffer from social anxiety and suicidal tendency. Moreover, adolescents might feel that they will not be understood by adults, and caregivers might oversee suicidal signs, given that suicidal tendencies are highly tabooed [76, 77].

Furthermore, the IDK responses were unevenly distributed across the ITQ-CG items with differences in PTSD versus the CPTSD-specific DSO items. Specifically, percentages tended to be higher for the first three PTSD items, and lower for the DSO items. The three PTSD items RE1 “Having upsetting dreams”, RE2 “Having powerful images and memories”, and AV1 “Avoiding internal reminders” reflecting internal processes, are likely less readily identified or understood, especially in the case of a child not being able to speak about these processes, be it because of a child age-bounded expression or because of non-disclosing. Items indicating psychological processes that are easier to understand, e.g. AV2 “Avoiding external reminders”, TH1 “Being ‘super-alert’, watchful or on guard” or TH2 “Feeling jumpy or easily startled” might be easier to disclose or better to identify for the caregiver having a close relationship with the child. Symptoms of intrusion or avoidance may not be observable for a caregiver or only present in response to a trauma trigger, e.g. when a caregiver was not present, or not informed by another person [78]. Issues such as the DSO items NSC1 “Feeling like a failure”, NSC2 “Feeling worthless”, or DR1 “Feeling distant or cut-off from people” might mirror the inability of a child to disclose these symptoms towards a caregiver or the caregiver’s reception and reflection of such a disclosure. However, some symptoms may be easier to observe (distant or cut-off from people) for closely related caregivers, as suggested by the prerequisite that the informing caregivers should share a close relationship and spend a substantial amount of time with the child [13, 79].

### “Knowers” versus ”don’t knowers”

The results of the LCA revealed a two-class solution between “Knowers” and “Don’t Knowers”, corroborating the rates of predicted “Knowers” (65%), versus predicted “Don’t Knowers” (35%) in this concrete sample.

Overall, this study deals with a sample of highly burdened children and thus concerned caregivers, since our data was collected at the entry assessment to a child psychiatry hospital. It can be expected that this group of caregivers might have a rather long history of consulting mental health experts and reporting symptoms. A systematic review [80] showed that, on average, adolescents and young adults saw 2.9 help-seeking contacts before receiving care. As family experiences with mental health care and exposure to mental health related information were found to be associated with caregivers‘ mental health literacy [21], it could be hypothesized that the caregivers in this study had a reasonable understanding of their children’s symptoms. Caregivers who are less experienced with the mental health care system may exhibit less mental health literacy. This underlines the need for public campaigns on mental health so that caregivers learn to better understand their children’s mental health symptoms and seek professional help if necessary.

Our study is associated with a long tradition of studies which provided evidence for the discrepancies between self-reports and caregiver-reports [6, 81]. Despite several models having been postulated, proposing multiple strategies to cope with the lack of knowledge about the discrepancies [25], no reduction in these discrepancies has been found thus far [6].

### Limitations and future directions

This study comes with certain limitations. First, it deals with a highly burdened sample, thus it is not possible to generalize the results to other less burdened samples. Second, no comparison group was given, nor was a research design applied to compare the ITQ-CG score between parents with and without IDK responses. Third, the caregiver group is probably biased by a dominance of female caregivers and is not coherent in its composition, as parents, grandparents, and institutional caregivers were part of the dyads. However, this diversity seems not to play a role with regard to using IDK responses. Overall, we had limited data about the caregivers, e.g. ethnic background or education, which is a further weakness of our study. Fourth, the ADES-8 was not designed for children younger than 11. Our self-report sample consisted predominantly of older children, however, 14.59% were younger than 11 at the time of the assessment. Unfortunately, there is no existing screening instrument for children younger than 11, therefore data on the ADES-8 need to be interpreted with caution. Furthermore, measurement error (e.g. random error) might have biased our results. Acknowledging that the measurement error can never be fully controlled, we used identical instruments for children and caregivers, in order to minimize this error.

Notably, most of the dyads of informants seemed to know each other well, almost 50% of the caregivers did not use the IDK option. Therefore, most of them likely have experience in understanding the meaning of the items, probably due to a long history in filling out questionnaires, screening tools and psychological tests. Most of the children entering a psychiatric hospital for in-house treatment usually passed several mental health examinations [21]. They likely underwent some treatment options and have seen, probably together with their caregivers, several mental health experts, so that the IDK responses might not be any more appropriate. Instead, a first-time presentation of a child for assessing psychological symptoms from the child-caregiver dyad might have been more adequate to better understand the function and value of the IDK responses. However, we did not have access to this group of children and caregivers. This would be a fruitful area for future work.

Moreover, further research aiming for a better insight into the knowledge of caregivers might reach out for a comparison of caregivers with a burdened child and dyads from the general population or with dyads which are for the first time confronted with reporting symptoms, so that possible training effects in symptom reporting would be avoided. Also, a comparison of ITQ-CG with and without using IDK responses might add to the understanding. A necessary next step should be considering relationship characteristics of caregiver-child dyads when examining discrepancies and agreements between these ratings. Future studies should also try to conduct a detailed assessment of caregivers’ sociodemographic characteristics and explore the association between these characteristics and caregivers’ knowledge about children’s psychological burden. Finally, to the best of our knowledge, the present study was the first one to investigate the utility of an IDK option in children’s trauma assessment. For a better understanding of challenges and opportunities regarding this option, more research is highly needed. Among others, future studies should compare the endorsement of the IDK response in items capturing internal vs. external processes. Moreover, associations between children’s symptoms and caregivers’ IDK responses should be more closely examined, as well as the possible influence of the IDK option on the concordance between child- and caregiver reports. Our study lays the groundwork for upcoming studies in this field of research.

## Conclusions

Clinically this study seems to be of relevance, since we work with data stemming from the practice as it is applied in a child psychiatry hospital. The discrepancies and the uneven distribution of IDK responses may remind us of the relevance of different information sources and the importance of carefully analyzing and interpreting them when shaping decisions for treatment and psychosocial support for the caregivers. Identifying symptoms that parents are more frequently not familiar with could be an important aspect for psychoeducation. Moreover, the association of social anxiety and suicidal tendencies with IDK responses points to the necessity of psychoeducation concerning these symptoms. Importantly, gender and age (except for the variable RE1, i.e. having upsetting dreams) were not associated with the frequency of the caregivers’ IDK responses in our sample. This stresses the need to support caregivers of children of all age groups and genders in detecting and reporting children’s mental health problems. Finally, IDK responses might guide clinicians in asking parents and children specifically, since discordances in children- and caregiver reports are also related with treatment adherence and treatment success [82].

## Data Availability

The data cannot be shared due to the child protection policy of the psychiatric hospital where the data were collected.

## Abbreviations

PTSD: Post-traumatic stress disorders
CPTSD: Post-traumatic stress disorders
DSM-5: Diagnostic and Statistical Manual of Mental Disorders, Fifth Edition
CATS: Child and Adolescent Trauma Screen
ICD-11: International Classification of Diseases 11th Revision
ITQ-CA: International Trauma Questionnaire– Child and Adolescent Version
ITQ-CG: International Trauma Questionnaire– Caregiver Version
IDK: I don‘t know
DSO: Disturbances in self-organization
RE 1, RE 2: Items assessing re-experiencing
AV 1, AV 2: Items assessing avoidance
TH 1, TH 2: Items assessing threat
AD 1, AD 2: Items assessing affective dysregulation
NSC 1, NSC 2: Items assessing negative self-concept
DR 1, DR 2: Items assessing disturbances in relationships
SMFQ: Short Mood and Feelings Questionnaire
GAD: Generalized anxiety disorder
PSC-17: Pediatric Symptom Checklist
ADES-8: Adolescent Dissociative Experiences Scale
GAD-7: Generalized Anxiety Disorder Scale
DSM-IV: Diagnostic and Statistical Manual of Mental Disorders, Fourth Edition
APA: American Psychiatric Association
LCA: Latent Class Analysis
AIC: Akaike Information Criterion
BIC: Bayesian Information Criterion
LMR: Lo-Mendell-Rubin adjusted likelihood ratio
CG: Caregivers

## References

[1] Copeland WE, Keeler G, Angold A, Costello EJ. Traumatic events and posttraumatic stress in childhood. Arch Gen Psychiat. 2007;64:577–84. 10.1001/archpsyc.64.5.577. 17485609 10.1001/archpsyc.64.5.577

[2] Alisic E, Zalta AK, van Wesel F, Larsen SE, Hafstad GS, Hassanpour K, Smid GE. Rates of post-traumatic stress disorder in trauma-exposed children and adolescents: meta-analysis. Br J Psychiatry. 2014;204:335–40. 10.1192/bjp.bp.113.131227. 24785767 10.1192/bjp.bp.113.131227

[3] Redican E, Hyland P, Cloitre M, McBride O, Karatzias T, Murphy J, et al. Prevalence and predictors of ICD-11 posttraumatic stress disorder and complex PTSD in young people. Acta Psychiatr Scand. 2022;146:110–25. 10.1111/acps.13442. 35503737 10.1111/acps.13442 PMC9540630

[4] Cook A, Spinazzola J, Ford J, Lanktree C, Blaustein M, Cloitre M, et al. Complex trauma in children and adolescents. Psychiatr Ann. 2005;35:390–8. 10.3928/00485713-20050501-05.

[5] McKay MT, Cannon M, Chambers D, Conroy RM, Coughlan H, Dodd P, et al. Childhood trauma and adult mental disorder: A systematic review and meta-analysis of longitudinal cohort studies. Acta Psychiatr Scand. 2021;143:189–205. 10.1111/acps.13268. 33315268 10.1111/acps.13268

[6] De Los Reyes A, Epkins CC. Introduction to the special issue. A dozen years of demonstrating that informant discrepancies are more than measurement error: toward guidelines for integrating data from multi-informant assessments of youth mental health. J Clin Child Adolesc Psychol. 2023;52:1–18. 10.1080/15374416.2022.2158843. 36725326 10.1080/15374416.2022.2158843

[7] de Los Reyes A, Thomas SA, Goodman KL, Kundey SMA. Principles underlying the use of multiple informants’ reports. Annu Rev Clin Psychol. 2013;9:123–49. 10.1146/annurev-clinpsy-050212-185617. 23140332 10.1146/annurev-clinpsy-050212-185617 PMC4103654

[8] Grills AE, Ollendick TH. Multiple informant agreement and the anxiety disorders interview schedule for parents and children. J Am Acad Child Adolesc Psychiatry. 2003;42:30–40. 10.1097/00004583-200301000-00008. 12500074 10.1097/00004583-200301000-00008

[9] Cooley DT, Jackson Y. Informant discrepancies in child maltreatment reporting: A systematic review. Child Maltreat. 2022;27:126–45. 10.1177/1077559520966387. 33054358 10.1177/1077559520966387

[10] de Los Reyes A, Kazdin AE. Informant discrepancies in assessing child dysfunction relate to dysfunction within Mother-Child interactions. J Child Fam Stud. 2006;15:643–61. 10.1007/s10826-006-9031-3. 21243074 10.1007/s10826-006-9031-3 PMC3020626

[11] Achenbach TM, McConaughy SH, Howell CT. Child/adolescent behavioral and emotional problems. Implications of cross-informant correlations for situational specificity; 1987. 3562706

[12] De Los Reyes A, Kazdin AE. Informant discrepancies in the assessment of childhood psychopathology: a critical review, theoretical framework, and recommendations for further study. Psychol Bull. 2005;131:483–509. 10.1037/0033-2909.131.4.483. 16060799 10.1037/0033-2909.131.4.483

[13] De Los Reyes A, Augenstein TM, Wang M, Thomas SA, Drabick DAG, Burgers DE, Rabinowitz J. The validity of the multi-informant approach to assessing child and adolescent mental health. Psychol Bull. 2015;141:858–900. 10.1037/a0038498. 25915035 10.1037/a0038498 PMC4486608

[14] Haselgruber A, Sölva K, Lueger-Schuster B. Symptom structure of ICD-11 complex posttraumatic stress disorder (CPTSD) in trauma-exposed foster children: examining the international trauma Questionnaire - Child and adolescent version (ITQ-CA). Eur J Psychotraumatol. 2020;11:1818974. 10.1080/20008198.2020.1818974. 33244361 10.1080/20008198.2020.1818974 PMC7678682

[15] Dyb G, Holen A, Brænne K, Indredavik MS, Aarseth J. Parent-child discrepancy in reporting children’s post-traumatic stress reactions after a traffic accident. Nord J Psychiatry. 2003;57:339–44. 10.1080/08039480310002660. 14522606 10.1080/08039480310002660

[16] Mai TA, Scheeringa MS. Caregiver and child agreement on traumatic events, PTSD, internalizing, externalizing, and ADHD problems in a child welfare population. J Public Child Welf. 2021;15:251–74. 10.1080/15548732.2019.1701612.

[17] Skar A-MS, Jensen TK, Harpviken AN. Who reports what? A comparison of child and caregivers´ reports of child trauma exposure and associations to post-traumatic stress symptoms and functional impairment in child and adolescent mental health clinics. Res Child Adolesc Psychopathol. 2021;49:919–34. 10.1007/s10802-021-00788-y. 33625640 10.1007/s10802-021-00788-y PMC8154822

[18] Wamser RA. Complex trauma and sexual abuse: relations to caregiver-child symptom disagreement. J Child Sex Abuse. 2023;32:793–812. 10.1080/10538712.2023.2257176. 10.1080/10538712.2023.2257176 37705222

[19] Woolgar F, Garfield H, Dalgleish T, Meiser-Stedman R. Systematic review and meta-analysis: prevalence of posttraumatic stress disorder in trauma-exposed preschool-aged children. J Am Acad Child Adolesc Psychiatry. 2022;61:366–77. 10.1016/j.jaac.2021.05.026. 34242737 10.1016/j.jaac.2021.05.026 PMC8885427

[20] Jorm AF, Korten AE, Jacomb PA, Christensen H, Rodgers B, Pollitt P. Mental health literacy: a survey of the public’s ability to recognise mental disorders and their beliefs about the effectiveness of treatment. Med J Aust. 1997;166:182–6. 10.5694/j.1326-5377.1997.tb140071.x. 9066546 10.5694/j.1326-5377.1997.tb140071.x

[21] Hurley D, Swann C, Allen MS, Ferguson HL, Vella SA. A systematic review of parent and caregiver mental health literacy. Community Ment Health J. 2020;56:2–21. 10.1007/s10597-019-00454-0. 31541315 10.1007/s10597-019-00454-0

[22] Cumsille P, Darling N, Martínez ML. Shading the truth: the patterning of adolescents’ decisions to avoid issues, disclose, or lie to parents. J Adolesc. 2010;33:285–96. 10.1016/j.adolescence.2009.10.008. 19926123 10.1016/j.adolescence.2009.10.008

[23] Kerr M, Stattin H, Burk WJ. A reinterpretation of parental monitoring in longitudinal perspective. J Res Adolescence. 2010;20:39–64. 10.1111/j.1532-7795.2009.00623.x.

[24] Duhig AM, Renk K, Epstein MK, Phares V. Interparental agreement on internalizing, externalizing, and total behavior problems: A meta-analysis. Clin Psychol Sci Pract. 2000;7:435–53. 10.1093/clipsy.7.4.435.

[25] De Los Reyes A. Strategic objectives for improving Understanding of informant discrepancies in developmental psychopathology research. Dev Psychopathol. 2013;25:669–82. 10.1017/S0954579413000096. 23880384 10.1017/S0954579413000096

[26] Martel MM, Pan PM, Hoffmann MS, Gadelha A, do Rosário MC, Mari JJ, et al. A general psychopathology factor (P factor) in children: structural model analysis and external validation through Familial risk and child global executive function. J Abnorm Psychol. 2017;126:137–48. 10.1037/abn0000205. 27748619 10.1037/abn0000205

[27] Sachser C, Berliner L, Holt T, Jensen TK, Jungbluth N, Risch E, et al. International development and psychometric properties of the child and adolescent trauma screen (CATS). J Affect Disord. 2017;210:189–95. 10.1016/j.jad.2016.12.040. 28049104 10.1016/j.jad.2016.12.040

[28] Sachser C, Berliner L, Risch E, Rosner R, Birkeland MS, Eilers R, et al. The child and adolescent trauma screen 2 (CATS-2) - validation of an instrument to measure DSM-5 and ICD-11 PTSD and complex PTSD in children and adolescents. Eur J Psychotraumatol. 2022;13:2105580. 10.1080/20008066.2022.2105580. 35928521 10.1080/20008066.2022.2105580 PMC9344962

[29] World Health Organisation. ICD-11 for Mortality and Morbidity Statistics: Disorders specifically associated with stress. 21.08.2024. https://icd.who.int/browse/2024-01/mms/en#991786158. Accessed 21 Aug 2024.

[30] Bruckmann P, Haselgruber A, Sölva K, Lueger-Schuster B. Comparing rates of ICD-11 and DSM-5 posttraumatic stress disorder in Austrian children and adolescents in foster care: prevalence, comorbidity and predictors. Eur J Psychotraumatol. 2020;11:1767988. 10.1080/20008198.2020.1767988. 33029314 10.1080/20008198.2020.1767988 PMC7473114

[31] Wang L, Fang R, Chen C, Cao C. A comparison of ICD-11 and DSM-5 criteria of PTSD among Chinese trauma-exposed adolescent samples. Front Psychiatry. 2023;14:1186138. 10.3389/fpsyt.2023.1186138. 37383620 10.3389/fpsyt.2023.1186138 PMC10293836

[32] Ho GWK, Liu H, Karatzias T, Hyland P, Cloitre M, Lueger-Schuster B, et al. Validation of the international trauma Questionnaire-Child and adolescent version (ITQ-CA) in a Chinese mental health service seeking adolescent sample. Child Adolesc Psychiatry Ment Health. 2022;16:66. 10.1186/s13034-022-00497-4. 35962396 10.1186/s13034-022-00497-4 PMC9375312

[33] Løkkegaard SS, Elklit A, Vang ML. Examination of ICD-11 PTSD and CPTSD using the international trauma Questionnaire - Child and adolescent version (ITQ-CA) in a sample of Danish children and adolescents exposed to abuse. Eur J Psychotraumatol. 2023;14:2178761. 10.1080/20008066.2023.2178761. 37052084 10.1080/20008066.2023.2178761 PMC9980161

[34] Lueger-Schuster B, Negrão M, Veiga E, Henley S, Rocha JC. The international trauma Questionnaire - Caregiver version. ITQ-CG); 2023.

[35] Haselgruber A, Weindl-Wagner D, Zagaria A, Zajec K, Noske J, Lueger-Schuster B. Construction and initial validation of the international trauma Questionnaire - Caregiver version (ITQ-CG): assessing ICD-11 PTSD and CPTSD in children from caregiver perspective. Eur J Psychotraumatol. 2025;16:2493025. 10.1080/20008066.2025.2493025. 40326422 10.1080/20008066.2025.2493025 PMC12057772

[36] Denman DC, Baldwin AS, Betts AC, McQueen A, Tiro JA. Reducing I don’t know responses and missing survey data: implications for measurement. Med Decis Making. 2018;38:673–82. 10.1177/0272989X18785159. 29962272 10.1177/0272989X18785159 PMC6076331

[37] Jokovic A, Locker D, Stephens M, Kenny D, Tompson B, Guyatt G. Measuring parental perceptions of child oral health-related quality of life. J Public Health Dent. 2003;63:67–72. 10.1111/j.1752-7325.2003.tb03477.x. 12816135 10.1111/j.1752-7325.2003.tb03477.x

[38] Oransky M, Hahn H, Stover CS. Caregiver and youth agreement regarding youths’ trauma histories: implications for youths’ functioning after exposure to trauma. J Youth Adolesc. 2013;42:1528–42. 10.1007/s10964-013-9947-z. 23580028 10.1007/s10964-013-9947-z

[39] Purdam K, Sakshaug J, Bourne M, Bayliss D. Understanding ‘Don’t know’ answers to survey questions - an International comparative analysis using interview paradata. Innovation: Eur J Social Sci Res. 2020;1–23. 10.1080/13511610.2020.1752631.

[40] Williamson V, Hiller RM, Meiser-Stedman R, Creswell C, Dalgleish T, Fearon P, et al. The parent trauma response questionnaire (PTRQ): development and preliminary validation. Eur J Psychotraumatol. 2018;9:1478583. 10.1080/20008198.2018.1478583. 29938010 10.1080/20008198.2018.1478583 PMC6008584

[41] Sargisson RJ, Stanley PG, Hayward A. Multi-informant scores and gender differences on the strengths and difficulties questionnaire for new Zealand children. New Z J Psychol. 2016;45.

[42] Davé S, Nazareth I, Senior R, Sherr L. A comparison of father and mother report of child behaviour on the strengths and difficulties questionnaire. Child Psychiatry Hum Dev. 2008;39:399–413. 10.1007/s10578-008-0097-6. 18266104 10.1007/s10578-008-0097-6

[43] Lam TCM, Green KE, Bordignon C. Effects of item grouping and position of the don’t know option on questionnaire response. Field Methods. 2002;14:418–32. 10.1177/152582202237730.

[44] Noske J. Unveröffentlichtes Arbeitspapier zu Versorgungszahlen der 3 KJPP Abteilungen in NÖ, lt. Einwohnerstatistik.; 29.02.2020.

[45] Haselgruber A, Weindl D, Zagaria A, Zajec K, Noske J, Lueger-Schuster B. Initial validation of the International Trauma Questionnaire– Caregiver version (ITQ-CG): Assessing ICD-11 PTSD and CPTSD from caregiver perspective: [Manuscript submitted for publication]. 2024. 10.1080/20008066.2025.2493025 PMC12057772 40326422

[46] Cloitre M, Bisson JI, Brewin CR, Hyland P, Karatzias T, Lueger-Schuster B, Shevlin M. International Trauma Questionnaire-Child and Adolescent Version (ITQ-CA); 2018. 10.1186/s13034-022-00497-4 PMC9375312 35962396

[47] Kazlauskas E, Zelviene P, Daniunaite I, Hyland P, Kvedaraite M, Shevlin M, Cloitre M. The structure of ICD-11 PTSD and complex PTSD in adolescents exposed to potentially traumatic experiences. J Affect Disord. 2020;265:169–74. 10.1016/j.jad.2020.01.061. 32090738 10.1016/j.jad.2020.01.061

[48] Angold A, Costello EJ, Messer SC, Pickles A. Development of a short questionnaire for use in epidemiological studies of depression in children and adolescents. Int J Methods Psychiatr Res,:237–49.

[49] Thapar A, McGuffin P. Validity of the shortened mood and feelings questionnaire in a community sample of children and adolescents: a preliminary research note. Psychiatry Res. 1998;81:259–68. 10.1016/s0165-1781(98)00073-0. 9858042 10.1016/s0165-1781(98)00073-0

[50] Birmaher B, Khetarpal S, Brent D, Cully M, Balach L, Kaufman J, Neer SM. The screen for child anxiety related emotional disorders (SCARED): scale construction and psychometric characteristics. J Am Acad Child Adolesc Psychiatry. 1997;36:545–53. 10.1097/00004583-199704000-00018. 9100430 10.1097/00004583-199704000-00018

[51] Thabrew H, Stasiak K, Bavin L-M, Frampton C, Merry S. Validation of the mood and feelings questionnaire (MFQ) and short mood and feelings questionnaire (SMFQ) in new Zealand help-seeking adolescents. Int J Methods Psychiatr Res. 2018;27:e1610. 10.1002/mpr.1610. 29465165 10.1002/mpr.1610 PMC6877137

[52] Gardner W, Murphy M, Childs G, Kelleher K, Pagano M, Jellinek M, et al. The PSC-17: A brief pediatric symptom checklist with psychosocial problem subscales. A report from PROS and ASPN. Ambul Child Health. 1997;5:225–36.

[53] Bergmann P, Lucke C, Nguyen T, Jellinek M, Murphy JM. Identification and utility of a short form of the pediatric symptom Checklist-Youth Self-Report (PSC-17-Y). Eur J Psychol Assess. 2020;36:56–64. 10.1027/1015-5759/a000486.

[54] Murphy JM, Bergmann P, Chiang C, Sturner R, Howard B, Abel MR, Jellinek M. The PSC-17: subscale scores, reliability, and factor structure in a new National sample. Pediatrics. 2016. 10.1542/peds.2016-0038. 27519444 10.1542/peds.2016-0038 PMC5005018

[55] Martínez-Taboas A, Shrout PE, Canino G, Chavez LM, Ramírez R, Bravo M, et al. The psychometric properties of a shortened version of the Spanish adolescent dissociative experiences scale. J Trauma Dissociation. 2004;5:33–54. 10.1300/J229v05n04_03.16957783

[56] Waller N, Putnam FW, Carlson EB. Types of dissociation and dissociative types: A taxometric analysis of dissociative experiences. Psychol Methods. 1996;1:300–21. 10.1037/1082-989X.1.3.300.

[57] Holmes EA, Brown RJ, Mansell W, Fearon RP, Hunter ECM, Frasquilho F, Oakley DA. Are there two qualitatively distinct forms of dissociation? A review and some clinical implications. Clin Psychol Rev. 2005;25:1–23. 10.1016/j.cpr.2004.08.006. 15596078 10.1016/j.cpr.2004.08.006

[58] Mossman SA, Luft MJ, Schroeder HK, Varney ST, Fleck DE, Barzman DH, et al. The generalized anxiety disorder 7-item scale in adolescents with generalized anxiety disorder: signal detection and validation. Ann Clin Psychiatry. 2017;29:227–A234. 29069107 PMC5765270

[59] American Psychiatric Association. Diagnostic and statistical manual of mental disorders fourth edition, text revision: DSM-IV-TR. Washington, DC: American Psychiatric Association; 2000.

[60] Tiirikainen K, Haravuori H, Ranta K, Kaltiala-Heino R, Marttunen M. Psychometric properties of the 7-item generalized anxiety disorder scale (GAD-7) in a large representative sample of Finnish adolescents. Psychiatry Res. 2019;272:30–5. 10.1016/j.psychres.2018.12.004. 30579178 10.1016/j.psychres.2018.12.004

[61] Beck AT, Steer RA, Brown G. Beck depression Inventory–II. BDI-II): APA PsycTests; 1996.

[62] Toledano-Toledano F, Contreras-Valdez JA. Validity and reliability of the Beck depression inventory II (BDI-II) in family caregivers of children with chronic diseases. PLoS ONE. 2018;13:e0206917. 10.1371/journal.pone.0206917. 30485299 10.1371/journal.pone.0206917 PMC6261561

[63] Green KL, Brown GK, Jager-Hyman S, Cha J, Steer RA, Beck AT. The predictive validity of the Beck depression inventory suicide item. J Clin Psychiatry. 2015;76:1683–6. 10.4088/JCP.14m09391. 26717528 10.4088/JCP.14m09391

[64] Lee E-H, Lee S-J, Hwang S-T, Hong S-H, Kim J-H. Reliability and validity of the Beck depression Inventory-II among Korean adolescents. Psychiatry Investig. 2017;14:30–6. 10.4306/pi.2017.14.1.30. 28096872 10.4306/pi.2017.14.1.30 PMC5240453

[65] Steer RA, Cavalieri TA, Leonard DM, Beck AT. Use of the Beck depression inventory for primary care to screen for major depression disorders. Gen Hosp Psychiatry. 1999;21:106–11. 10.1016/s0163-8343(98)00070-x. 10228890 10.1016/s0163-8343(98)00070-x

[66] R Core Team. R: A Language and environment for statistical computing. Vienna, Austria: R Foundation for Statistical Computing; 2023.

[67] McDonald JH, editor. Handbook of biological statistics. 3rd ed. Sparky House Publishing; 2014.

[68] Linzer DA, Lewis JB. PoLCA: an R package for polytomous variable latent class analysis. J Stat Softw. 2011. 10.18637/jss.v042.i10.

[69] Lo Y, Mendell NR, Rubin DB. Testing the number of components in a normal mixture. Biometrika. 2001;88:767–78. 10.1093/biomet/88.3.767.

[70] Bauer J. A primer to latent profile and latent class analysis. In: Goller M, Kyndt E, Paloniemi S, Damşa C, editors. Methods for researching professional learning and development: challenges, applications and empirical illustrations. Springer International Publishing; 2022. pp. 243–68.

[71] Bulteel K, Wilderjans TF, Tuerlinckx F, Ceulemans E. CHull as an alternative to AIC and BIC in the context of mixtures of factor analyzers. Behav Res Methods. 2013;45:782–91. 10.3758/s13428-012-0293-y. 23307573 10.3758/s13428-012-0293-y

[72] Kuha JAIC. Comparisons of assumptions and performance. Sociol Methods Res. 2004;33:188–229. 10.1177/0049124103262065.

[73] Remschmidt H. Psychosocial milestones in normal puberty and adolescence. Horm Res. 1994;41(Suppl 2):19–29. 10.1159/000183955. 8088699 10.1159/000183955

[74] Colich NL, Hanford LC, Weissman DG, Allen NB, Shirtcliff EA, Lengua LJ, et al. Childhood trauma, earlier pubertal timing, and psychopathology in adolescence: the role of corticolimbic development. Dev Cogn Neurosci. 2023;59:101187. 10.1016/j.dcn.2022.101187. 36640624 10.1016/j.dcn.2022.101187 PMC9842860

[75] Gallagher M, Prinstein MJ, Simon V, Spirito A. Social anxiety symptoms and suicidal ideation in a clinical sample of early adolescents: examining loneliness and social support as longitudinal mediators. J Abnorm Child Psychol. 2014;42:871–83. 10.1007/s10802-013-9844-7. 24390470 10.1007/s10802-013-9844-7 PMC5496444

[76] Kushal SA, Amin YM, Reza S, Shawon MSR. Parent-adolescent relationships and their associations with adolescent suicidal behaviours: Secondary analysis of data from 52 countries using the Global School-based Health Survey. EClinicalMedicine. 2021. 10.1016/j.eclinm.2020.100691 10.1016/j.eclinm.2020.100691 PMC7846673 33554083

[77] da Silva Filho OC, de Souza Minayo MC. Triple taboo: considerations about suicide among children and adolescents. [Triplo tabu: sobre o suicídio na infância e na adolescência]. Cien Saude Colet. 2021;26:2693–8. 10.1590/1413-81232021267.07302021. 34231682 10.1590/1413-81232021267.07302021

[78] Scheeringa MS. PTSD in children younger than the age of 13: toward developmentally sensitive assessment and management. Journ Child Adol Trauma. 2011;4:181–97. 10.1080/19361521.2011.597079. 10.1080/19361521.2011.597079 PMC6379904 30792828

[79] Achenbach TM. As others see Us. Curr Dir Psychol Sci. 2006;15:94–8. 10.1111/j.0963-7214.2006.00414.x.

[80] MacDonald K, Fainman-Adelman N, Anderson KK, Iyer SN. Pathways to mental health services for young people: a systematic review. Soc Psychiatry Psychiatr Epidemiol. 2018;53:1005–38. 10.1007/s00127-018-1578-y. 30136192 10.1007/s00127-018-1578-y PMC6182505

[81] Haselgruber A, Sölva K, Lueger-Schuster B. Perspective matters: differences between child- and caregiver-reports of emotion regulation mediating the relationship between cumulative childhood trauma and mental health problems in foster children. Curr Issues Child Sex Abuse Gend Health Outcomes -Part II. 2020;107:104558. 10.1016/j.chiabu.2020.104558. 10.1016/j.chiabu.2020.104558 32559554

[82] Wamser-Nanney R, Campbell CL. Factors associated with Caregiver-Child symptom concordance among Trauma-Exposed children. Child Maltreat. 2021;26:152–61. 10.1177/1077559520927472. 32462927 10.1177/1077559520927472

